# Self-Evaluation of Decision-Making: A General Bayesian Framework for Metacognitive Computation

**DOI:** 10.1037/rev0000045

**Published:** 2017-01

**Authors:** Stephen M. Fleming, Nathaniel D. Daw

**Affiliations:** 1 Wellcome Trust Centre for Neuroimaging, University College London; 2 Princeton Neuroscience Institute and Department of Psychology, Princeton University

**Keywords:** computation, confidence, decision-making, metacognition

## Abstract

People are often aware of their mistakes, and report levels of confidence in their choices that correlate with objective performance. These metacognitive assessments of decision quality are important for the guidance of behavior, particularly when external feedback is absent or sporadic. However, a computational framework that accounts for both confidence and error detection is lacking. In addition, accounts of dissociations between performance and metacognition have often relied on ad hoc assumptions, precluding a unified account of intact and impaired self-evaluation. Here we present a general Bayesian framework in which self-evaluation is cast as a “second-order” inference on a coupled but distinct decision system, computationally equivalent to inferring the performance of another actor. Second-order computation may ensue whenever there is a separation between internal states supporting decisions and confidence estimates over space and/or time. We contrast second-order computation against simpler first-order models in which the same internal state supports both decisions and confidence estimates. Through simulations we show that second-order computation provides a unified account of different types of self-evaluation often considered in separate literatures, such as confidence and error detection, and generates novel predictions about the contribution of one’s own actions to metacognitive judgments. In addition, the model provides insight into why subjects’ metacognition may sometimes be better or worse than task performance. We suggest that second-order computation may underpin self-evaluative judgments across a range of domains.

People are often aware of their mistakes, and report levels of confidence in their choices that correlate with objective performance. These assessments of decision quality are important for the guidance of behavior, particularly when external feedback is absent or sporadic, and such metacognitive abilities are particularly well-developed in humans (Beran, Brandl, Perner, & Proust, 2012; Metcalfe, 1996; Norman & Shallice, 1986; Shea et al., 2014). Understanding the relationship between self-evaluations and performance is a key goal for multiple interlocking research areas including judgment and decision-making (Lichtenstein, Fischhoff, & Phillips, 1982), education (Veenman, Wilhelm, & Beishuizen, 2004), social psychology (Heatherton, 2011), consciousness science (Lau & Rosenthal, 2011), and clinical disorders (David, Bedford, Wiffen, & Gilleen, 2012; Goldstein et al., 2009). However, an appropriate computational framework that subsumes both confidence and error detection is lacking (Yeung & Summerfield, 2012). In addition, accounts of dissociations between performance and metacognition have often relied on ad hoc assumptions, precluding a unified account of intact and impaired metacognition.

In the laboratory, the mechanisms underpinning self-evaluation of performance have been investigated by asking subjects to judge their confidence in simple decisions. As we will outline in further detail below, decision confidence can be defined as a subjective probability of a decision being correct (Aitchison et al., 2015; Pouget et al., 2016), and is one of many forms of uncertainty that the brain may encode (Bach & Dolan, 2012; Meyniel, Schlunegger, & Dehaene, 2015). Decision confidence can be elicited through a variety of measures including self-reports, postdecision wagers, and opt-out responses (see Kepecs & Mainen, 2012, for a review), and previous studies show that variability in decision confidence tracks changes in objective performance (Henmon, 1911; Nelson & Narens, 1990; Peirce & Jastrow, 1885) and supports the recognition of task errors (Gehring, Goss, Coles, Meyer, & Donchin, 1993; Rabbitt, 1966; Rabbitt & Rodgers, 1977; Yeung, Botvinick, & Cohen, 2004).

Formal models of decision confidence have focused on the role played by the decision variable—an internal subjective state that is influenced by incoming sensory evidence (Kepecs, Uchida, Zariwala, & Mainen, 2008; Kiani & Shadlen, 2009; Merkle & Van Zandt, 2006; Ratcliff & Starns, 2009; Vickers, 1979). For instance, in signal detection theoretic models, the absolute distance of the decision variable from a criterion is a proxy for confidence (Cartwright & Festinger, 1943; Ferrell & McGoey, 1980; Kepecs et al., 2008; Macmillan & Creelman, 2005; Suantak, Bolger, & Ferrell, 1996; Treisman & Faulkner, 1984). Dynamic extensions of signal detection theory accumulate evidence for or against a particular choice (Link & Heath, 1975; Gold & Shadlen, 2002), and several variants of this approach have linked the state of the decision variable at decision time to confidence (Kiani & Shadlen, 2009; Merkle & Van Zandt, 2006; Moreno-Bote, 2010; Ratcliff & Starns, 2009; Vickers, 1979; see Fetsch, Kiani, & Shadlen, 2015; Yeung & Summerfield, 2012 for reviews). Empirically, putative neural correlates of decision variables are also correlated with subjective confidence (De Martino, Fleming, Garrett, & Dolan, 2013; Gherman & Philiastides, 2015; Kiani & Shadlen, 2009; Komura, Nikkuni, Hirashima, Uetake, & Miyamoto, 2013; Zizlsperger, Sauvigny, Händel, & Haarmeier, 2014).

However, a close coupling between decision variables and confidence is potentially in tension with a burgeoning literature identifying dissociations between performance and metacognition. There are systematic differences between factors affecting task performance and confidence in perceptual decisions, including attentional or stimulus manipulations (Bona & Silvanto, 2014; Graziano & Sigman, 2009; Lau & Passingham, 2006; Rahnev et al., 2011; Vlassova, Donkin, & Pearson, 2014; Wilimzig, Tsuchiya, Fahle, Einhäuser, & Koch, 2008), individual differences (Baird, Cieslak, Smallwood, Grafton, & Schooler, 2015; Barttfeld et al., 2013; Fleming, Weil, Nagy, Dolan, & Rees, 2010; McCurdy et al., 2013; Song et al., 2011), developmental trajectory (E. C. Palmer, David, & Fleming, 2014; L. G. Weil et al., 2013) and brain lesions or reversible inactivation in both humans (Del Cul, Dehaene, Reyes, Bravo, & Slachevsky, 2009; Fleming, Ryu, Golfinos, & Blackmon, 2014; Rounis, Maniscalco, Rothwell, Passingham, & Lau, 2010), nonhuman primates (Komura et al., 2013), and rodents (Lak et al., 2014). In addition, psychiatric and neurological disorders are often associated with impairments in self-evaluation (David et al., 2012; Fleming et al., 2014; Goldstein et al., 2009; Pannu & Kaszniak, 2005; Schmitz & Johnson, 2007; Weiskrantz, Warrington, Sanders, & Marshall, 1974).

Such dissociations may arise for a number of reasons. First, the evidence contributing to decisions may be subject to further processing that introduces additional variability into confidence reports. This further processing may occur over space and/or time. For instance, metacognitive reports may require a neural “read-out” of confidence from decision circuitry (Insabato, Pannunzi, Rolls, & Deco, 2010; Maniscalco & Lau, 2012; Shimamura, 2000). Alternatively, confidence may be affected by continued processing of predecision evidence in time (Baranski & Petrusic, 1998; Moran, Teodorescu, & Usher, 2015; Rabbitt & Vyas, 1981; Resulaj, Kiani, Wolpert, & Shadlen, 2009; S. Yu, Pleskac, & Zeigenfuse, 2015) or the receipt of new postdecision evidence (Bronfman et al., 2015; Kvam, Pleskac, Yu, & Busemeyer, 2015; Navajas, Bahrami, & Latham, 2016). Second, evidence contributing to decisions may be inaccessible to confidence reports. A canonical example is blindsight, in which cortically blind individuals may perform visual discrimination tasks well above chance but be unable to self-evaluate their performance, having a poor impression of whether they performed well or badly on individual trials (Ko & Lau, 2012; Persaud, McLeod, & Cowey, 2007; Persaud et al., 2011; Schmid et al., 2010; Weiskrantz, 1998; Weiskrantz et al., 1974). Third, evidence contributing to confidence reports may be inaccessible to decision-making. A classic example of this phenomenon is error detection, in which human subjects rapidly signal errors made in simple laboratory tasks (Rabbitt, 1966; Rabbitt & Rodgers, 1977). The presence of the “error-related negativity” (ERN) in the scalp EEG signal around the time of the response is consistent with a rapid evaluation that one’s impending response is likely to be incorrect (Gehring et al., 1993). Together these findings suggest an architecture in which evidence supporting decisions and confidence is maintained at least partly separately and in parallel (Baranski & Petrusic, 2001; Charles, King, & Dehaene, 2014; Del Cul et al., 2009; Ro, Shelton, Lee, & Chang, 2004; Schmid et al., 2010).

This variety of performance-confidence dissociations has hitherto precluded a unified account of metacognition in decision-making. Here we set out to account for such dissociations in a general framework in which confidence operates as a second-order computation about one’s own performance. Our core proposal is that within a single individual, samples of sensory evidence underpinning decisions and confidence judgments are distinct but coupled. Such a distinction between decision and confidence variables arises necessarily in many of the situations considered above, and once this is formally recognized, sound statistical inference differs in key ways from that prescribed by first-order signal detection theory (Green & Swets, 1966). In our analysis, self-evaluation of decision performance is achieved by leveraging the confidence sample and one’s own actions to infer the performance of the coupled decision system, over time and/or space. We develop these ideas in a Bayesian ideal observer model, at Marr’s computational level, jumping off from the standard signal detection theory framework that has served as the foundation for much work in perception and metacognition. These more abstract computational considerations would, of course, be complimented by more implementational considerations at Marr’s other levels of analysis, as indeed has proved a highly synergistic program in the case of signal detection theory and its real-time generalizations such as the sequential likelihood ratio test (Gold & Shadlen, 2002; Link & Heath, 1975).

It will turn out that this framework, inspired by the dissociations reviewed above, holds key implications for metacognitive computation in general. First, second-order computation naturally accommodates different behavioral manifestations of metacognition such as confidence and error detection within a common framework. The intuition, which will be formalized below, is that a secondary view on the decision problem is required for a system to view itself in error (Charles et al., 2014; James, 1950; Pasquali, Timmermans, & Cleeremans, 2010; Rabbitt, 1966). Error monitoring and confidence have typically been studied in separate literatures (Yeung & Summerfield, 2012), but here a continuum of confidence ranging from being certain of committing an error to being sure of being correct emerges naturally from the model architecture. Second, a second-order account predicts that one’s own actions will contribute to self-evaluation. The intuition here is that rather than actions simply signaling the output of a decision pathway, they may themselves carry information about the subject’s internal states that is otherwise inaccessible to confidence reports.

In the sections that follow we compare the qualitative predictions of second-order computation to those made by first-order accounts with and without postdecisional processing, and evaluate these predictions against the empirical literature on decision confidence and error monitoring. We will show that first-order models are special cases of second-order computation that arise under particular noise conditions (see Figure 1). Our analysis thus clarifies the situations in which these simpler architectures are suitable, and the sorts of approximations being made by adopting them when these conditions are not satisfied. We go on to demonstrate how a second-order perspective accounts for individual differences in metacognitive bias and accuracy, and may explain cases in which metacognition is sometimes better than task performance. We close by outlining the implications of this framework for future empirical studies and discuss possible neural implementations of second-order computation.[Fig-anchor fig1]

## Model Overview

We consider three classes of model of how a subject generates a report of confidence in his or her decision. All models have the same basic ingredients. First, we define a categorical world state, *d*, such as whether a stimulus is moving left (*d* = −1) or right (*d* = 1). Second, the subject makes a response *a* to indicate their perceived state of the world (i.e., left, *a* = −1, or right, *a* = 1). On each “trial” internal states *X* = [*X_act_* *X_conf_*] denote the decision and confidence variables. To make a decision, the subject chooses “right” if *X_act_* > 0, and left otherwise:

$$a=\{\begin{array}{c} 1\;\;\text{if}\;\;X_{act}>0\\ -1\;\;\;\text{otherwise} \end{array}$$ 1

We define the subject’s confidence *z* as a degree of belief that a particular choice was correct (i.e., choice *a* reflected the true state of the world *d*), given a particular set of internal states *X*, model *m* and model parameters ϑ:

z=P(a=d|X,a,ϑ,m) 2

In all model simulations we assume Gaussian noise for how internal states *X* are generated from world states *d*. However, the models differ in how these states are coupled, and how confidence is computed, as described in the following sections.

### First-Order Model

In the simplest “first-order” model we assume that the decision and confidence variables are identical, such that the same internal state supports both choices and confidence. First, the decision variable *X_act_* is obtained from a Gaussian distribution conditional on the world state:

$$X_{act}\;∼\;\;N(d,\;\sigma^{2})$$ 3

The confidence variable *X_conf_* = *X_act_*. Confidence is then a transformation of the posterior belief in *d* conditional on the action taken (or equivalently, the sign of *X_act_*):

$$z=P\left(a=d|X_{conf},\;a,\;\sigma^{2}\right)=\{\begin{array}{c} P\left(d=1|X_{conf},\sigma^{2}\right)\;\text{if}\;a=1\\ 1-P\left(d=1|X_{conf},\sigma^{2}\right)\;\text{if}\;a=-1 \end{array}$$ 4

where Bayes’ rule provides the posterior probability of a particular world state (assuming flat priors on *d*):

$$P\left(d|X_{conf},\sigma^{2}\right)=\frac{P(X_{conf}|d,\sigma^{2})}{\sum_{d}P(X_{conf}|d,\sigma^{2})}$$ 5

### Postdecisional Model

In the postdecisional model, the confidence variable *X_conf_* is derived from *X_act_* plus additional information about the world state, *X_new_*:

$$X_{conf}\;=X_{act}\;+X_{new}$$ 6

For ease of exposition we define *X_new_* as an additional sample of evidence^1^, *X_new_* ∼ *N*(*d*, σ^2^). One can imagine different generative models—the key property here is that the true world state *d* is conditionally independent from the action *a* (and its decision variable *X_act_*), given the confidence decision variable *X_conf_*. Informally, *X_conf_* should provide all the information contained in *X_act_*. This will be satisfied, for instance, if *X_act_* and *X_conf_* are both states of a perfect accumulator (with *X_conf_* read out later, see, e.g., Resulaj et al., 2009; van den Berg et al., 2016), but not if the accumulator is lossy or if *X_conf_* arises from a noisy readout of *X_act_*, degrading the signal with additional noise.

The observer then derives confidence in a similar fashion to the first-order model above:

$$z=\;P\left(a=d|X_{conf},\;a,\;2\sigma^{2}\right)=\{\begin{array}{c} P\left(d=1|X_{conf},2\sigma^{2}\right)\;\text{if}\;a=1\\ 1-P\left(d=1|X_{conf},2\sigma^{2}\right)\;\text{if}\;a=-1 \end{array}$$ 7

Note that the first-order model is a special case of the postdecisional model when *X_act_* = *X_conf_*.

### Second-Order Model

The second-order model is subtly but importantly different from the first-order and postdecisional models. Unlike in the first-order case, confidence is not derived directly from *X_conf_* – instead *X_conf_* is leveraged, together with the observed action *a* and knowledge of the covariance between *X_conf_* and *X_act_*, to infer the state of the decider at the time of choice.

We first describe a second-order model of confidence in another individual’s performance to provide the intuition for the within-subject case, and to demonstrate the symmetry between evaluating one’s own actions and those of another actor. Consider two individuals, an Actor (*act*) and Confidence-rater (*conf*). The actor is carrying out a two-choice discrimination task as described above. Both receive internal samples *X_act_* and *X_conf_* generated from binary world state *d* (e.g., a stimulus moving left or right). We model these samples as draws from a bivariate Gaussian with covariance matrix ∑:

$$\left[\begin{array}{c} X_{act}\\ X_{conf} \end{array}\right]\;∼\;N(d,\sum )$$ 8

$$\sum =\left[\begin{array}{cc} \sigma_{act}^{2} & \rho \sigma_{act}\sigma_{conf}\\ \rho \sigma_{act}\sigma_{conf} & \sigma_{conf}^{2} \end{array}\right]$$ 9

The covariance matrix has 3 parameters: σ_*act*_, σ_*conf*_, and ρ. σ_*act*_ and σ_*conf*_ control the noise of the signal for the Actor and the Confidence-rater, respectively. The correlation parameter ρ governs the association between the two samples: capturing, for instance, the fact that the variance in the two observers’ samples of the stimulus will be partly common (attributable to objective variation in the stimulus) and partly distinct (attributable, e.g., to distinct sensory and neural noise). The Confidence-rater’s job is to say how confident she is in the Actor responding correctly, or the posterior probability that the Actor’s action *a* was appropriate for the inferred state of the world *d*, conditional on beliefs about different sources of variability. To do this, the observer infers (for the purpose of marginalizing) the state of the decision variable driving choice (*X_act_*) from the confidence variable (*X_conf_*):

$$z=P\left(a=d|X_{conf},a,\sum \right)=\{\begin{array}{c} P\left(d=1|X_{conf},\;a,\sum \right)\;\;\text{if}\;a=1\\ 1-P\left(d=1|X_{conf},\;a,\sum \right)\;\;\text{if}\;a=-1\; \end{array}$$ 10

where *P*(*d*|*X_conf_*, *a*, ∑) ∝ *P*(*d*|*X_conf_*, ∑)*P*(*a*|*X_conf_*, *d*, ∑)

$$=P\left(d|X_{conf},\;\sum \right)\;\int P\left(a|X_{act},\sum \right)P(X_{act}|X_{conf},d,\sum )\;dX_{act}$$ 11

**Figure 1 fig1:**
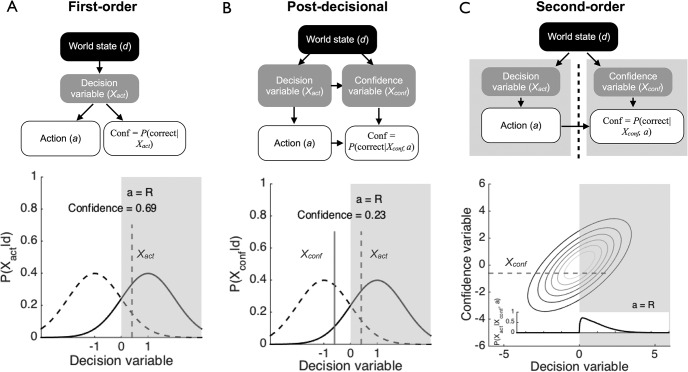
Schematic graphical models of self-evaluation. Upper panels show graphical models (with variance/covariance parameters omitted for clarity). In each model, a categorical world state (e.g., stimulus = left [−1] or right [1]) gives rise to a binary action (left or right). Building on signal detection theory, we assume both stimuli give rise to internal decision variables that are Gaussian distributed along a unitary decision axis. To make an action, the observer choose “right” if the decision variable is greater than 0, and “left” otherwise. Lower panels depict a computation of confidence on a single trial of each model, in which the observer responds “right”. (A) First-order model. The world state generates a decision variable *X_act_* that supports both actions and confidence reports. (B) Postdecisional first-order model. As in (A), but allowing the confidence variable (*X_conf_*) to sample additional evidence about the world state, which in this case leads to recognition of an error (confidence < 0.5). (C) Second-order model. The decision and confidence variables are represented as two correlated hidden states. A computation of decision confidence proceeds by first inferring the distribution of possible decision variables conditional on the confidence variable (shown by the probability distribution in the inset), and marginalizing conditional on the subject’s action to arrive at an appropriate confidence level.

The core of our proposal is that individuals generate confidence in their own performance by applying an analogous computation to their own actions (Figure 1C). Importantly, in Equation 10 the probability of being correct is determined not only by *X_conf_* but also one’s own action *a* and beliefs about the fidelity of the decision and confidence variables, captured by ∑. In other words, second-order inference reflects an active process of inferring the state of the decider, rather than a passive sensitivity to the difficulty of the decision. In Appendix A we derive analytic solutions to this equation for two-choice decision scenarios assuming Gaussian noise.

**Figure 2 fig2:**
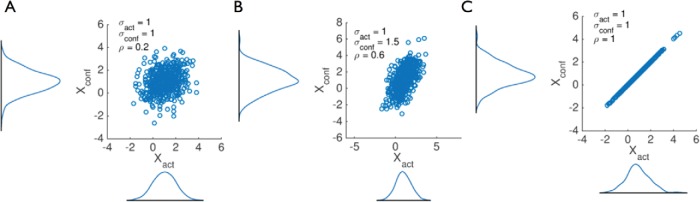
Illustration of effects of second-order model parameters on decision and confidence variables. Each panel shows samples of the decision variable (*X_act_*) and the confidence variable (*X_conf_*) drawn from models with different parameter settings. The correlation coefficient ρ increases from (A) to (C). Panel (B) shows the effect of selectively increasing the variability in the confidence variable (compare the width of the marginal distributions of *X_conf_* and *X_act_*). The parameter settings in panel (C) mimic a first-order model in which *X_act_* and *X_conf_* are identical. See the online article for the color version of this figure.

In the between-subjects case, we might expect limited correlation between the confidence and decision variables, as depicted in Figure 2A. In the within-subject case, this correlation may be higher, although one evidence stream may be noisier than the other, thereby weakening the information that either the Actor or the Confidence-rater has about the true world state (Figure 2B). The model architecture is agnostic about how the relationship between *X_act_* and *X_conf_* arises: it may be that they remain segregated in the brain (e.g., in parallel pathways); *X_conf_* may depend on the same neural activity as *X_act_* at a later time point, or *X_conf_* may reflect a noisy read-out of *X_act_*. The many possible relationships between *X_act_* and *X_conf_* are flexibly accommodated via the parameters of the covariance matrix ∑. In the special case in which ρ = 1 and σ_*act*_ = σ_*conf*_, the second-order model reduces to the first-order case, as on any given trial the same evidence supports both actions and confidence (Figure 2C).[Fig-anchor fig2]

We note that these model variants are naturally nested, with each representing an extension of the previous case. The first-order model is a special case of the postdecisional model in which the decision and confidence variables are identical, and the postdecisional model is a special case of the second-order model in which *X_conf_* is a sufficient statistic for *X_act_* with respect to *d* (e.g., when evidence is accumulated without forgetting). Indeed, variants of the first-order or postdecisional models outlined above are optimal under limited cases in which the confidence computation has direct access to the actor’s decision variable. However, the computational considerations we highlight here apply to all but the simplest cases in which internal states underpinning performance are transparently accessible to those underpinning confidence.

## Results (1): Features of Second-Order Computation

In this section we describe qualitative features of first- and second-order computation, and relate these to key findings in the empirical literature.

### Relationship Between Decision Confidence, Accuracy, and Stimulus Strength

**Figure 3 fig3:**
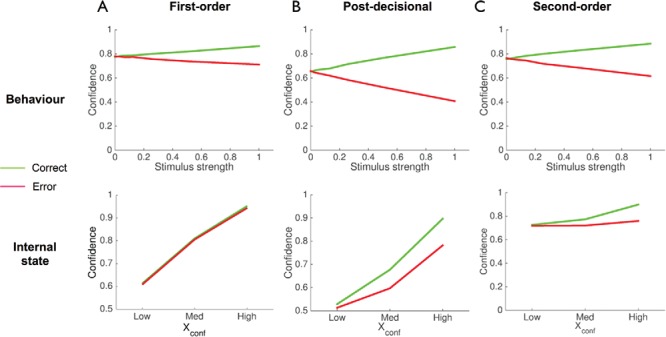
Internal representations supporting decision confidence. Simulations of first-order (A), postdecisional (B), and second-order (C) models showing how confidence changes as a function of stimulus strength and decision accuracy. The upper panels show confidence as a function of objective stimulus strength; the lower panels show confidence as a function of the internal state of each model. See the online article for the color version of this figure.

We begin with internal representations supporting decision confidence. Decision confidence typically increases with stimulus evidence for correct judgments, but decreases with stimulus evidence for errors (“X-pattern”, Figure 3; Kepecs et al., 2008; Lak et al., 2014; Sanders et al., 2016; although see Kiani et al., 2014). Here we show that all three model variants are able to reproduce this pattern, and therefore observing an X-pattern in behavior is not diagnostic of first- or second-order computation.[Fig-anchor fig3]

### First-Order Model

To simulate confidence as a function of stimulus strength we modified all models such that the sample mean depends on stimulus strength θ (varying between 0 and 1; μ = *d*θ; see Appendix B for details of this and other simulations). The upper panel in Figure 3A shows that the first-order model reproduces the qualitative X-pattern observed in the behavioral data despite the confidence and decision variables being identical. The intuition for this pattern is as follows. A given direction *d* and stimulus strength θ leads to a range of samples *X_act_*, and the possibility of erroneous responses. As θ increases, the likely values of |*X_conf_*| (=|*X_act_*|) following an incorrect response therefore decrease in magnitude. To take a concrete example, suppose we have a leftward trial (*d* = −1). If the subject’s sample *X_act_* is + 0.05, she will erroneously respond “right” and derive confidence from a monotonic transformation of |*X_conf_*|. But this subjective sample may have arisen from many different objective stimulus strengths θ, including both correct and error trials, and occur more often with some than others. When the experimenter then plots the subject’s confidence as a function of the externally manipulated variable θ, a divergent pattern of confidence emerges for correct and error trials. In other words, the X-pattern is due to the necessity of relating observed confidence to θ (which is unknown to the subject) rather than to *X_conf_* (which is unknown to the experimenter).

However, if it were possible to determine the decision variable on individual trials, we would predict that confidence always scales monotonically with |*X_conf_*| for both correct and error trials in the first-order case (Figure 3A, lower panel). The internal state representation of a first-order model does not show the X-pattern.^2^

### Postdecisional Model

The same X-pattern is obtained for confidence derived from simulations of the postdecisional model (Figure 3B). However, in this case the model’s internal state diverges as a function of choice accuracy attributable to cases in which the decision and confidence variables dissociate (cf. Figure 1B). In other words, if it were possible for the experimenter to know *X_conf_* on a single trial, a postdecisional account would predict divergent relationships between confidence and *X_conf_* on correct and error trials (Figure 3B, lower panel).

### Second-Order Model

Finally, the behavioral X-pattern also emerges from a second-order computation of confidence, but for different reasons (Figure 3C). Here the model detects its own errors by applying second-order inference. Specifically, given a sample *X_conf_*, the model generates a probability that its action matched the most likely state of the world. In this case, confidence decreases on error trials with increasing θ because there tends to be increasing evidence (from *X_conf_*) that the action taken was inappropriate. As in the postdecisional model, an interaction with choice accuracy is also observed in the model’s internal state (Figure 3C, lower panel).

In summary, all models are able to account for the X-pattern relating confidence to stimulus strength as a function of accuracy, but do so for different reasons. The pattern emerges in the first-order model because of an imprecise mapping between the experimenter-observed variable θ and internal state *X_act_*; it emerges in the post-decisional and second-order models because of the effect exerted by beliefs counter to one’s choice on the posterior probability of having made a correct action. The internal states of the postdecisional and second-order models also show an X-pattern.

### Relationship Between Confidence and Error Detection

Human subjects are able to rapidly detect errors made in simple laboratory tasks (Rabbitt, 1966; Rabbitt & Rodgers, 1977). Other work has investigated the dynamics of changes of mind—a switch from an initial, often erroneous response to an alternative, correct response (Resulaj et al., 2009). Both error detection and changes of mind can be formalized as a subjective probability of success for a chosen action being lower than that of an alternative action, which in two-choice discrimination corresponds to a decision confidence level less than 0.5.

**Figure 4 fig4:**
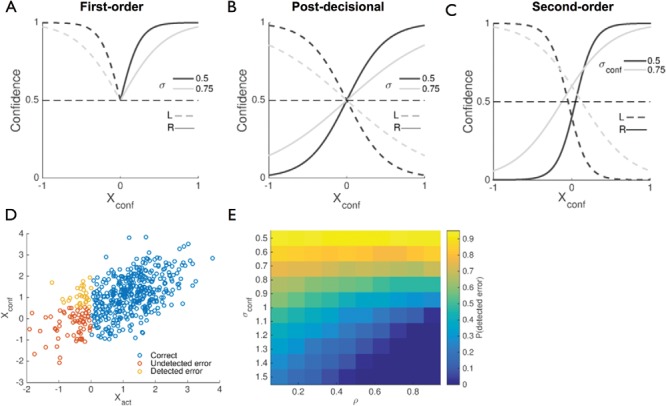
Internal representations supporting error detection. (A) Confidence as a function of the decision variable and uncertainty parameter σ in the first-order model. (B, C) Confidence as a function of the confidence variable, chosen action and uncertainty parameter σ_*conf*_ in the postdecisional model (B) and second-order model (C). (D) Simulation of how error detection emerges from correlated samples in the second-order model. Samples are generated from a true world state *d* = 1 with parameter settings σ_*act*_ = 1, σ_*conf*_ = 1 and ρ = 0.6. The model makes errors when *X_act_* falls to the left of the neutral (0) criterion. A subset of these objective errors are “detected” due to the confidence variable providing evidence that the alternative action is preferred, generating a confidence level of less than 0.5. (D) Heat map revealing how the proportion of detected errors in (C) varies according to model parameters σ_*conf*_ and ρ. Objective accuracy (governed by σ_*act*_) is constant. See the online article for the color version of this figure.

It is notable that such representations are precluded in the simplest first-order model because the same evidence drives both choices and confidence, resulting in a lower bound on confidence of 0.5 (Figure 4A). In other words, if a single decision variable indicates that the alternative option is preferable, then the action also follows suit; dissociations between actions and confidence do not occur and confidence is monotonic in |*X_conf_*|. In contrast, in both the postdecisional and second-order models (Figure 4B, C), confidence maps out a space from being sure that an error has been committed to being sure of a correct response, due to regimes in which the model infers that its action *a* was at odds with the most probable direction *d*, and there is no longer a monotonic mapping between |*X_conf_*| and confidence. Finally, Figure 4C illustrates a feature of second-order computation that we will return to below: even when the confidence variable provides equivocal evidence about the world (*X_conf_* = 0), the model’s confidence is not necessarily at chance (0.5). Instead, for the parameters used in this simulation, confidence when *X_conf_* = 0 is around 0.7, due to the confidence computation also incorporating knowledge about the average reliability of actions, that is, σ_*act*_ (Drugowitsch, Moreno-Bote, & Pouget, 2014). In summary, postdecisional and second-order models are able to reproduce error-detection-like behavior (*P*(correct) < 0.5), but the simplest first-order model cannot.[Fig-anchor fig4]

The internal representations of the second-order model that support error detection are illustrated in Figure 4D. Here we sampled moderately correlated samples of *X_act_* and *X_conf_* from world state *d* = 1 (i.e., the true stimulus class is “right”). By applying a neutral decision criterion, the observer erroneously responds “left” whenever *X_act_* is less than zero. However, whether this error will be detected depends on whether *X_conf_* provides enough (positive) evidence in support of the alternative, correct response (orange samples in Figure 4D). The proportion of detected errors is itself governed by the covariance of *X_conf_* and *X_act_*. Figure 4E simulates the proportion of detected errors for a constant performance level (σ_*act*_ = 1; ∼84% correct). Error detection is highest when σ_*conf*_ is low, because the confidence variable provides accurate information about the true world state. Notably error detection also depends on the correlation between the samples—as ρ approaches 1 (lower right quadrant of the heatmap) the model reduces to the first-order case and error detection is again precluded.

These simulations of error detection are of course an oversimplification—the criterion for whether to report an error is itself under subject control, and may be adjusted above or below 0.5 in the face of changing incentives (Neyman & Pearson, 1933; Steinhauser & Yeung, 2010). The aim here is simply to show that both postdecisional and second-order models naturally handle error detection and changes of mind by modeling cases in which the confidence and decision variables disagree.

### Influences of Self-Generated Actions on Confidence

**Figure 5 fig5:**
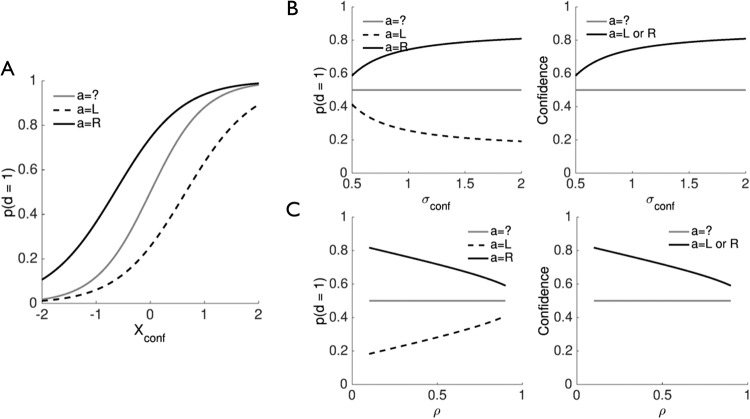
Influence of choices on second-order model confidence. (A) Posterior probability of a rightward world state as a function of confidence variable *X_conf_* and the chosen action. (B, C) The lefthand panels show the influence of actions on the posterior probability of *d* = 1 for a constant, uninformative sample (*X_conf_* = 0). The righthand panels show the corresponding confidence level. In all panels gray lines show expected confidence from a first-order model for comparison. (B) As the confidence variable becomes less informative (σ_*conf*_ increases), actions have a greater effect on posterior beliefs. (C) As the correlation between *X_act_* and *X_conf_* increases, actions provide less new information about the possible values of d, and their influence on confidence reduces. Constant parameters in all panels are set at σ_*act*_ = 1, σ_*conf*_ = 1, ρ = 0.4.

A counterintuitive but important feature of second-order computation is that one’s own actions may causally affect subsequent confidence ratings, particularly if *X_act_* and *X_conf_* are only weakly coupled. This influence arises because actions carry information about the subject’s internal states, leading a rational observer to incorporate her own actions as additional data when computing confidence. Consider Figure 5A. Plotted on the *y* axis is the posterior probability that the current world state is rightward (*d* = 1) as a function of confidence variable *X_conf_*. Intuitively, as *X_conf_* becomes more positive, the model gains greater evidence that *d* = 1. However, having taken an action *a*, this inference is modulated, such that a leftward action reduces the belief in rightward world states, whereas a rightward action boosts it.[Fig-anchor fig5]

To further explore this effect, we simulated the model’s confidence after “clamping” *X_conf_* at 0. In the first-order case (gray line in Figure 5B and 5C), the model is equivocal about the world state and confidence remains at 0.5. However, after an action is made, the second-order model leverages this new information to modulate its belief in *d*. The extent to which this modulation occurs is dependent on (beliefs about) the covariance of *X_act_* and *X_conf_*. As the confidence variable becomes more noisy (σ_*conf*_ increases), the information provided by *X_conf_* is less reliable and actions are given more weight (Figure 5B). Conversely, as the correlation between *X_act_* and *X_conf_* increases (ρ increases), actions provide less new information about the possible values of *d*, and the modulation of confidence by action decreases (Figure 5C).

**Figure 6 fig6:**
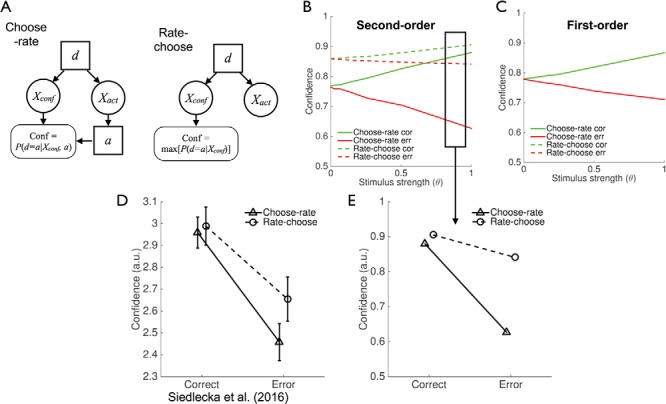
Predicted effects of choice on confidence. (A) Graphical models for choose-rate and rate-choose experiments illustrating the influence of actions on confidence in the choose-rate condition. (B) Simulation of confidence from choose-rate and rate-choose experiments as a function of stimulus strength and decision accuracy for the second-order model (σ_*act*_ = 1, σ_*conf*_ = 1, ρ = 0.6). Overall confidence (bias) decreases relative to the rate-choose condition when choices are made before confidence ratings (choose-rate), whereas the difference in confidence between correct and error trials (metacognitive sensitivity) increases. (C) As in (B) for the first-order model (σ_*act*_ = 1). Here the predictions for confidence from the choose-rate and rate-choose models are identical and the dotted lines are obscured. (D) Data replotted from Siedlecka et al. (2016), with permission, in which choice and rating order were manipulated. (E) Simulations of second-order model predictions at constant stimulus strength, plotted using same conventions as (D). See the online article for the color version of this figure.

**Figure 7 fig7:**
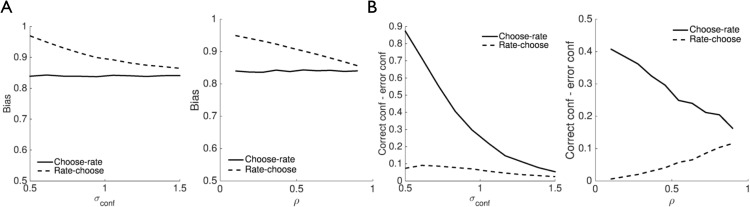
Effects of choice on confidence across a range of second-order model parameter settings. (A) Plots of bias as a function of model parameters σ_*conf*_ (left panel) and ρ (right panel). Across a range of parameter settings confidence is decreased in the choose-rate condition. In the σ_*conf*_ simulation, ρ = 0.6, whereas in the ρ simulation, σ_*conf*_ = 1. (B) Similar to (A) for metacognitive sensitivity (the difference between correct and error confidence). Across a range of parameter settings metacognitive sensitivity is increased in the choose-rate condition.

This feature of the model leads to a counterintuitive empirical prediction: elicitation of actions should affect confidence judgments. For instance, if subjects are asked to rate their confidence *before* their response (i.e., confidence in making a hypothetical response), then they may compute their confidence without conditioning on self-action (which is precluded in this case unless subjects covertly choose and then rate; Figure 6A). This leads to two effects (Figure 6B). First, the difference in confidence between correct and error trials should be greater (metacognitive sensitivity should increase) when ratings are given after a decision than before, due to the additional diagnostic information provided by the action. Second, ratings given after a decision should be systematically lowered compared to those given before (Figures 7A and B show that these qualitative effects are obtained across a large range of second-order model parameters). In contrast, actions do not provide any additional diagnostic information about hidden states in first-order accounts, and in the absence of additional postdecision evidence, confidence levels are equivalent whether elicited pre- or postdecision (Figure 6C).[Fig-anchor fig6][Fig-anchor fig7]

Empirical observation of a pattern similar to that depicted in Figure 6B would therefore provide support for a second-order model of confidence. While revising our manuscript for publication (and after developing these simulations) we became aware of a published dataset that directly tested and confirmed our predictions (Figure 6D). Siedlecka et al. (2016) asked subjects to provide confidence ratings about whether a target word presented on the screen was the solution to a previously studied anagram. In a between-subjects design, participants were assigned to one of three conditions: deciding if a target word was an anagram and then judging confidence (target-decision-metacognitive judgment, tDM); judging confidence after seeing the target but before making a decision (tMD); or rating confidence before seeing the target word (MtD). Here we focus on the difference between the tDM and tMD conditions, as they represent direct analogues of our choose-rate and rate-choose simulations. In Figure 6D we replot their data alongside the second-order model simulation at constant stimulus strength (Figure 6E). Siedlecka et al. (2016) found that metacognitive sensitivity was greater in the tDM than the tMD conditions, in accordance with the predictions of a second-order model in which actions inform confidence ratings. In addition, confidence was overall lower in the choose-rate case, although unlike the effect on metacognitive sensitivity, this was not statistically significant. As can be seen by comparing Figure 6D and E, the second-order model simulation qualitatively captures the patterns observed in Siedlecka et al.’s experiment.

### Conclusions

In this section we have explored features of first- and second-order models of confidence, and compared their qualitative predictions against empirical findings on confidence and error detection. We find that although all models can reproduce relationships between stimulus strength, accuracy, and confidence, only postdecisional and second-order models permit levels of confidence that may support error detection, and only a second-order account naturally accommodates findings that actions themselves influence confidence judgments. These results are summarized in Table 1.[Table-anchor tbl1]

## Results (2): Dissociations Between Performance and Confidence

### Modeling Dissociations Between Performance and Confidence

Metacognitive accuracy refers to the relationship between self-evaluation and performance, and comprises two components: sensitivity and bias^3^ (Fleming & Lau, 2014). Metacognitive sensitivity refers to the extent to which a subject can discriminate correct from incorrect performance on a first-order task, and can be assessed with Type II receiver operating characteristic (ROC) analysis (Clarke, Birdsall, & Tanner, 1959; Galvin, Podd, Drga, & Whitmore, 2003) or meta-*d*′, which indexes metacognitive sensitivity in units of decision *d*′ (Maniscalco & Lau, 2012, 2014). The logic is that if an observer has good sensitivity, she will be able to discriminate between her own correct and incorrect responses through offering up suitable confidence reports—lower confidence when incorrect, and higher confidence when correct. Metacognitive bias is the tendency to give higher overall confidence ratings, all else being equal. Note that bias is potentially independent of sensitivity—a subject might have high overall confidence but be unable to discriminate between correct and error trials.

In this section we show that second-order computation naturally accommodates changes in metacognitive sensitivity and bias through alterations in covariance parameters and beliefs about covariance parameters (hyperparameters), respectively, and handles cases in which metacognitive sensitivity is either better or worse than performance.

### Metacognitive Sensitivity

**Figure 8 fig8:**
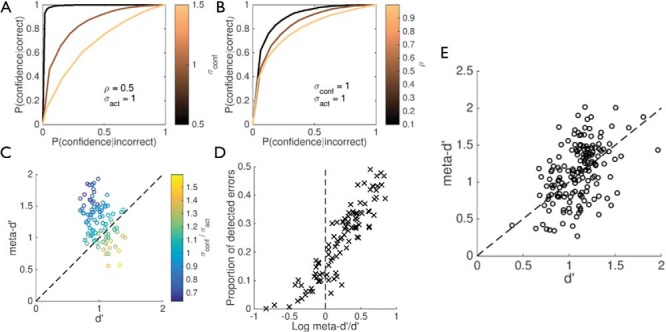
Modeling changes in metacognitive sensitivity in a second-order framework. (A) Simulated Type II ROCs for different levels of noise in the confidence variable, σ_*conf*_. As *X_conf_* becomes more variable, metacognitive sensitivity is reduced despite task performance remaining constant. (B) Simulated Type II ROCs for different levels of ρ. As the correlation between the confidence and decision variables is increased, metacognitive sensitivity is decreased. (C) Relationship between d′ and meta-d′ of simulated data sets color-coded by settings of model parameters σ_*conf*_ and σ_*act*_ (ρ = 0.5). Cases of “hyper”-metacognitive sensitivity in which meta-*d′* > *d′* are associated with parameter ratios less than 1, indicating greater variability in the decision variable compared to the confidence variable. (D) Relationship between meta-*d′*/*d′* of simulated data sets and proportion of detected errors in each dataset. Cases of meta-*d′*/*d′* > 1 (log(meta-*d′*/*d′*) > 0) are associated with an increase in the number of detected errors. E) Plot of *d′* against meta-*d′* obtained from data pooled across a number of empirical studies (Fleming et al., 2010; Fleming, Huijgen, & Dolan, 2012; E. C. Palmer et al., 2014; L. G. Weil et al., 2013), demonstrating the substantial frequency of hyper-metacognitive sensitivity observed in these data sets. See the online article for the color version of this figure.

Two distinct (but not mutually exclusive) sets of parameter changes may lead to reductions in the second-order model’s metacognitive sensitivity. In the first, metacognitive sensitivity is impoverished (Type II ROC area is reduced) as the noise in the confidence variable σ_*conf*_ is increased (Figure 8A). In the second, σ_*conf*_ remains constant but the correlation between *X_conf_* and *X_act_* is increased, leading to decreased metacognitive sensitivity despite task performance remaining constant (Figure 8B). In other words, while the precision of the confidence variable remains constant, increased coupling between the confidence and decision variables reduces the model’s ability to detect when its behavior may have been inappropriate (cf. Figure 4D).[Fig-anchor fig8]

### Accounting for Hyper- and Hypo-Metacognitive Sensitivity

In signal detection theoretic approaches to metacognition, Type I performance provides a theoretical upper bound on the Type II ROC (Galvin et al., 2003). In other words, it is not possible, under these accounts, for more signal to be available to the Confidence-rater than available to the Actor. Maniscalco and Lau provided an elegant method for comparing metacognitive sensitivity and performance by characterizing metacognitive sensitivity in units of Type I *d*′, which they label meta-*d*′ (Maniscalco & Lau, 2012). In this approach, an ideal observer’s meta-*d*′ equals *d*′, or the ratio meta-*d*′/*d*′ = 1. Suboptimal or hypo-metacognitive sensitivity results in values of meta-*d*′/*d*′ < 1 (Barrett, Dienes, & Seth, 2013; Maniscalco & Lau, 2014). Maniscalco and Lau suggested that empirical values of meta-*d*′/*d*′ > 1 (“hyper”-metacognitive sensitivity) may be attributable to artifacts of estimation error or criterion variability. But in our experience, such values are routinely observed in empirical studies (see Figure 8E), and recent work has highlighted that in certain circumstances hyper-metacognitive sensitivity may be more common than previously assumed (Charles, Van Opstal, Marti, & Dehaene, 2013; Scott, Dienes, Barrett, Bor, & Seth, 2014).

Building on the simulations of error detection considered above, we can understand how hyper-metacognitive sensitivity may naturally arise as a consequence of postdecisional and/or second-order computation. If the confidence variable provides additional valid information about the world state (in the second-order model, when ρ < 1 and σ_*conf*_ is low), the model reliably detects its own errors (Figures 4D, 8A and 8B). This may lead to circumstances in which metacognition is “better” than performance, that is, meta-*d*′ > *d*′. To demonstrate this we randomly sampled simulated data sets generated from a particular combination of σ_*act*_ (*d*′) and σ_*conf*_ (holding ρ constant at 0.5), and fitted meta-*d*′ to each dataset. Figure 8C plots *d*′ against meta-*d*′, color-coded according to the ratio of model parameters σ_*conf*_/σ_*act*_. It can be seen that when this ratio is small, values of meta-*d*′ > *d*′ are routinely obtained. Furthermore, when we interrogate the relationship between the proportion of detected errors (i.e., errors with confidence <0.5), hyper-metacognitive sensitivity is associated with the emergence of error detection in the model (Figure 8D). These results demonstrate that both hypo- and hyper-metacognitive sensitivity are accommodated by a second-order framework.

### Bias/Calibration

Up until now we have assumed that the covariance parameters associated with internal states are identical to those entering into the model inversion step when computing confidence. This is presumably an oversimplification. Instead, a subject’s beliefs (hyperparameters) about these parameters may be malleable, leading to systematic over- or underconfidence (Adams, Stephan, Brown, Frith, & Friston, 2013; Drugowitsch et al., 2014), and potentially accounting for systematic biases in self-evaluation.

**Figure 9 fig9:**
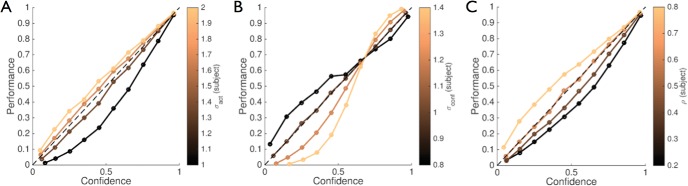
Modeling changes in metacognitive bias in a second-order framework. Simulated performance levels conditioned on 10 equally spaced confidence bins for different beliefs about parameters (A) σ_*act*_, (B) σ_*conf*_, or (C) ρ. In each panel we manipulated beliefs about the relevant parameter while holding the other two parameters constant. For all simulations the actual parameters used to generate samples were fixed at σ_*act*_ = 1.5, σ_*conf*_ = 1, ρ = 0.6. See the online article for the color version of this figure.

To illustrate how changing hyperparameters leads to bias, in Figure 9 we plot the model’s aggregate performance (proportion correct) conditioned on 10 levels of confidence for different settings of beliefs about parameters σ_*act*_, σ_*conf*_ and ρ. Importantly, for all simulations the actual parameters used to generate internal samples and decisions were fixed at σ_*act*_ = 1.5, σ_*conf*_ = 1, ρ = 0.6. The deviation of the curves from the identity line show that subtly different beliefs about the true underlying parameters are sufficient to produce a range of patterns of systematic over- or underconfidence, typical of the probability distortions observed in the experimental literature (Drugowitsch et al., 2014; Harvey, 1997; Zhang & Maloney, 2012).[Fig-anchor fig9]

## Discussion

We have proposed that metacognitive judgments of decision-making may depend on second-order computation about behavior, computationally equivalent to inferring the performance of another actor. A key insight is that as soon as one recognizes a distinction between the decision variable controlling behavior, versus the information guiding the confidence judgment, then except in special cases, correctly judging confidence requires inferring the causes of one’s own behavior. This general formalism subsumes several cases in which the internal states underlying performance and confidence may differ, such as dissociations over space and time. Second-order computation accounts for different behavioral manifestations of metacognition such as confidence and error detection within a single computational scheme. Furthermore, by positing coupled hidden states, a second-order framework naturally handles dissociations between performance and metacognition.

Nested within a second-order framework are simpler first-order accounts. We find that while first-order models can reproduce the empirical interrelationship of confidence, stimulus strength, and accuracy, only postdecisional and second-order models reproduce confidence levels that support error detection, and only the second-order model accommodates findings that actions themselves influence confidence judgments. Thus while we do not wish to propose that second-order computation always underpins confidence reports, some features of empirical data are at least consistent with the operation of second-order computation in a subset of cases. Although intentionally broad in scope, a second-order framework nevertheless makes concrete empirical predictions, including the influence of actions upon decision confidence and the commonality between neural mechanisms supporting confidence and error detection. Here we consider in greater detail how our model relates to previous models of error detection and confidence, and explore possible neural implementations of second-order computation.

### Relationship to Previous Models of Metacognition in Decision-Making

#### Models of error detection

A second-order framework suggests that errors are detected as a mismatch between an inference on the world state and the selected action. This approach is consistent with earlier accounts of error monitoring that emphasize the comparison between intentions and actions (Charles et al., 2014; Coles, Scheffers, & Holroyd, 2001; Holroyd & Coles, 2002; Holroyd, Yeung, Coles, & Cohen, 2005; Rabbitt & Rodgers, 1977). Although initially this literature focused on binary error signaling, there has been increasing recognition that similar principles may also underpin graded confidence judgments (Boldt & Yeung, 2015; Scheffers & Coles, 2000; Yeung & Summerfield, 2012). One influential model of error detection suggests that activation of two competing responses leads to conflict (and associated activation in the anterior cingulate cortex), and this conflict triggers the detection of an impending error (Yeung et al., 2004). An alternative perspective is that error detection relies instead on computing the likelihood of an error occurring in a given context (Alexander & Brown, 2011; Brown & Braver, 2005). The current framework provides a potential bridge between these accounts—error detection relies on “conflict” between two streams of evidence (see Figure 4C), but rather than the model signaling this conflict per se, it harnesses this disagreement to infer a probability that an error will occur.

Holroyd and colleagues proposed a neural network model of error detection which assigned value to state-action conjunctions by reinforcement learning (Holroyd et al., 2005; Holroyd & Coles, 2002). Once the model has been trained, actions that are inappropriate for a given state became associated with negative values, leading to a negative prediction error (and associated error-related negativity) at the time of response. This scheme also shares commonalities with second-order computation in that confidence is conditional on both state and action variables. However, it differs in that second-order computation does not explicitly represent stimulus–response conjunctions. Instead such associations are implicit in inverting a generative model of action when evaluating one’s performance.

#### Models of confidence

Several previous models of confidence have built upon evidence accumulation models of decision-making, accounting for key interrelationships between choice, confidence and response time (De Martino et al., 2013; Kiani & Shadlen, 2009; Kiani, Corthell, & Shadlen, 2014; Merkle & Van Zandt, 2006; Pleskac & Busemeyer, 2010; Ratcliff & Starns, 2009; Vickers, 1979). One instance where decoupling of information underlying decision and confidence arises is when a single representation of decision evidence evolves over time, as in our postdecisional model simulations. This idea—a sort of bridge along the way from first to second-order models—has been used to model confidence and changes of mind (Moran et al., 2015; Pleskac & Busemeyer, 2010; Resulaj et al., 2009; van den Berg et al., 2016), and can also be seen as a special case of the framework we present here. (In particular, as we discuss further below, our analysis indicates that even in this first-order-like case, a confidence judgment should be informed by the chosen action, unless the accumulation is perfect and without decay). We note the relationship between decision time and confidence is likely to be complicated, and dependent on the task and goal of the observer (Koizumi et al., 2015; Pleskac & Busemeyer, 2010). However, a compelling avenue for future work is to unfold second-order computation in time, propagating multiple hidden states, just as the drift-diffusion model represents a temporal unfolding of classical signal detection (Ratcliff, 1978). Initial work along these lines has explored how the propagation of multiple internal decision variables holds promise for unifying accounts of decisions and subjective reports (Del Cul et al., 2009; Fuss & Navarro, 2013; Kvam et al., 2015; Zandbelt, Purcell, Palmeri, Logan, & Schall, 2014). Such models may provide computational insights not only into the dynamics of self-evaluation, but also the evaluation of the decisions of others (Patel, Fleming, & Kilner, 2012).

There is ongoing debate over whether confidence computation is best accommodated by serial or parallel architectures (Fleming & Dolan, 2012; Maniscalco & Lau, 2014; Pleskac & Busemeyer, 2010). Maniscalco and Lau found that a signal detection model in which confidence is derived from a noisy hierarchical representation of evidence supporting a choice provided a better fit to rating data than alternatives in which evidence for choices and confidence evolved in parallel (Maniscalco & Lau, 2016). Similarly, Pleskac & Busemeyer’s 2-stage dynamic signal detection (2DSD) model proposes that a decision variable continues accumulating beyond the decision time, at which point confidence is determined by its relation to a set of response criteria (Pleskac & Busemeyer, 2010). This model accounts for a number of relationships between decision time, postdecision time and confidence. However, serial accumulation may not be sufficient to account for cases in which error detection is very fast, consistent with a parallel representation of evidence against the decision (Charles et al., 2014; Rabbitt, 1966). Del Cul and colleagues suggested that information for decisions and subjective reports is accumulated in parallel, and this architecture was able to mimic a selective alteration in subjective reports due to prefrontal brain damage (Del Cul et al., 2009).

A second-order approach offers a broader perspective on this debate, subsuming several special cases. Specifically, depending on the covariance of the model’s internal states, confidence ratings may appear to be determined by a hierarchical or parallel architecture. For instance, if σ_*act*_ < σ_*conf*_ and ρ is high, the model will appear hierarchical, in that confidence depends on the same evidence as actions, albeit with added noise. Conversely, if ρ is low, the model operates in a parallel fashion, and as σ_*act*_ approaches zero, cases of “blind insight” may occur in which the model is aware of making erroneous or correct actions despite performing at or near chance (Scott et al., 2014). Finally, there may be domains or tasks in which confidence reports show a particularly high degree of sophistication in tracking task performance, which would suggest that decision and confidence variables are tightly coupled, with little opportunity for dissociations (e.g., Barthelme & Mamassian, 2010; Meyniel, Sigman, & Mainen, 2015; Peters & Lau, 2015).

A further implication of second-order computation is that common mechanisms should support both confidence judgments and monitoring of errors. Most previous work on error monitoring has focused on discrete cases in which actions diverge from intentions under time pressure. The canonical finding is that an error-related negativity (ERN) originating in the anterior cingulate cortex is observed time-locked to the onset of the erroneous response (Dehaene, Posner, & Tucker, 1994; Gehring et al., 1993). In contrast, studies of confidence have tended to focus on cases in which perceptual uncertainty is manipulated but response requirements are trivial (although see Faisal & Wolpert, 2009; Fleming, Maloney, & Daw, 2013). There is now increasing recognition that multiple sources of variability affect the strength of error- and confidence signals in the brain; for instance, neural signatures of error detection are also modulated by the degree of sensory uncertainty of the subject (Charles et al., 2013; Navarro-Cebrian, Knight, & Kayser, 2013; Scheffers & Coles, 2000). In support of this idea, Boldt and Yeung recently provided direct evidence for a common neural substrate for confidence and error detection. By applying multivariate decoding analyses to EEG data recorded during a visual discrimination task, they showed that neural markers of error detection were also predictive of varying levels of confidence in correct choices (Boldt & Yeung, 2015).

### Varieties of Metacognitive Inaccuracy

The ability to discriminate one’s own correct and incorrect responses can be quantified by Type II ROC analysis (Clarke et al., 1959; Galvin et al., 2003; Maniscalco & Lau, 2012, 2014). Recently Maniscalco and Lau developed an elegant measure of metacognitive sensitivity, meta-*d*′, that quantifies the Type II ROC area in units of first-order *d*′ (Maniscalco & Lau, 2012). As shown in Figure 8, there may be a number of reasons for low meta-*d*′ in the current framework. Increased noise in the confidence variable may impair inference on world states and therefore impair classification of correct or incorrect responses. Conversely, an increase in correlation between the decision and confidence variables may lead to impaired insight, due to the model not being able to “recognize” when it may have been in error.

It is instructive to contrast the signal detection model underpinning meta-*d*′ with the Bayesian framework outlined here. Whereas meta-*d*′ is primarily a tool for estimating metacognitive sensitivity, second-order computation provides an underlying generative model for confidence and an explanatory framework for different types of dissociation between performance and confidence. In addition, whereas confidence in the meta-*d*′ model is specified in arbitrary units, second-order computation models decision confidence as a probability, thus allowing specification of parameters determining not only metacognitive sensitivity but also the extent of over- or underconfidence. It is therefore useful to view meta-*d*′ as complementary to our framework. Just as *d*′ provides a bias-free measure of perceptual sensitivity that depends on a number of underlying processes, meta-*d*′ provides a summary of an individual’s metacognitive sensitivity that is determined by the joint contribution of internal states and the computations applied to those states.

Multiple drivers of metacognitive sensitivity are also recognized by the stochastic detection and retrieval model (SDRM) of confidence in memory (Jang et al., 2012), which assumes that two samplings of evidence occur per stimulus, one leading to memory retrieval, and the other leading to a confidence rating. One important difference between second-order computation and the SDRM is that in the former, decision confidence is a probability of success derived from inverting a generative model of action, whereas in the latter, confidence is generated by comparing samples to additional criterion parameters. An intriguing consequence is that in the SDRM, an increase in ρ leads to increased metacognitive sensitivity, due to a tighter association between confidence and performance, whereas in second-order computation, an increase in ρ leads to a decrease in sensitivity, due to the model being unable to see itself in error (Figures 3D and 8B). Empirical work combined with model comparison could test these predictions.

Our model accommodates dissociations between decision-making and metacognition through alterations in the precision and coupling of internal states, such as the decision and confidence variables. However it is also possible that decision-making and metacognition have different inferential goals, and may be differentially sensitive to different types of information. Introducing these normative constraints into models of metacognition is an important goal for future work. For instance, it would be of interest to explore whether differential sensitivity to evidence for or against a choice (Koizumi et al., 2015; Maniscalco et al., 2016; Zylberberg et al., 2012), and differential effects of attention on performance and confidence (Rahnev et al., 2011; Solovey et al., 2015) could be accommodated in a Bayesian framework with appropriate constraints. The current framework may also provide a benchmark from which to assess other apparent suboptimalities in confidence that are normative when appropriate computational considerations are taken into account (e.g., the effects of actions on subsequent confidence ratings). Finally, we have shown that mismatches between the subject’s beliefs (hyperparameters) about different sources of uncertainty and the true parameters can lead to systematic over- and underconfidence (Adams et al., 2013; Drugowitsch et al., 2014), and thus potentially account for variability across individuals in metacognitive bias. How such hyperparameters are learnt over time is an important topic for future investigation.

### Influence of Choices on Confidence Judgments

A counterintuitive feature of second-order computation is that actions influence subsequent confidence ratings, all else being equal. This influence arises because actions contribute information about possible world states, leading a rational observer to incorporate his own actions as additional data when computing confidence (cf. Bem, 1967). This feature of the model has several empirical implications. A practical implication is that it pays to be cautious when comparing data from studies in which confidence is elicited with or without a preceding action. Several behavioral paradigms have been developed for eliciting decision confidence in both humans and nonhuman animals (Kepecs & Mainen, 2012). In retrospective judgment paradigms, an action intervenes between the stimulus and the confidence rating whereas in opt-out and simultaneous-report paradigms, confidence is elicited in parallel to or instead of the decision itself. Measures of confidence from these paradigms are often taken to be equivalent. However the current model predicts subtle differences in the role played by actions in retrospective judgment designs where the subject’s own responses may contribute additional evidence to the computation of confidence. Although perhaps counterintuitive, this is rational under the model architecture: to the extent that the confidence and decision variable have partially distinct information, the subject may gain additional information about the world state by “observing” her own actions.

A second-order framework makes concrete predictions about the effect of choices on confidence ratings—namely a decrease in overall confidence and an increase in sensitivity. In addition to the results of Siedlecka et al. (2016) that we document in Figure 6, other recent findings are consistent with these predictions. Manipulating the order of identification responses and subjective awareness ratings (including confidence and visibility scales) revealed increases in metacognitive sensitivity when identification responses preceded the rating (Wierzchoń et al., 2014). Zehetleitner and Rausch (2013) similarly compared first-order subjective ratings of a stimulus with second-order confidence in a previous decision, and found that the latter was associated with greater metacognitive sensitivity. Finally, Kvam and colleagues compared a choice with a no-choice (arbitrary mouse click) condition in a random-dot motion discrimination task (Kvam et al., 2015). They found that confidence judgments were less extreme and more accurate in the choice compared to the no-choice condition (see also Ronis & Yates, 1987; Sniezek et al., 1990 for similar findings); however, in this case effects of choice were modeled as interfering with a second stage of evidence accumulation, as sensory evidence continued to be available after the decision was made. Finally, in a recent study we tested for the influence of action-specific information on confidence in a near-threshold visual discrimination task by applying single-pulse TMS to the premotor cortex (Fleming et al., 2015). When stimulation was incongruent with the subjects’ actions, confidence judgments on correct trials were decreased, whereas congruent stimulation led to increased confidence. Performance remained unchanged. This pattern is potentially consistent with a contribution of action information to second-order computation.

The role of action in a second-order framework also reveals subtleties in the relationship between confidence and visibility judgments. In consciousness studies, confidence ratings are often considered proxies for perceptual awareness (Peirce & Jastrow, 1885). For instance, King and Dehaene (2014) suggest that within a signal detection framework, visibility is equivalent to assessing confidence in a detection response, and their model is able to account for several classical characteristics of conscious and unconscious perception. However, to the extent that subjects are applying second-order computation to assess their confidence in their response, we might observe that subjects leverage the information content of the response itself to inform their confidence ratings. For instance, blindsight patients with lesions to visual cortex may nevertheless develop a “hunch” that their response was correct, without acknowledging the existence of a corresponding visual conscious experience (Persaud et al., 2011). As described above, similar effects may also lead to changes in visibility ratings following responses in psychophysics experiments in healthy observers (Wierzchon et al., 2014). More broadly, these considerations suggest subtleties in inferring perceptual awareness from confidence ratings about the observer’s response, and alternative approaches for determining perceptual awareness may be preferred, such as forced-choice discrimination of stimulus visibility (Peters & Lau, 2015).

We note that there are certain cases in which one would *not* expect an influence of action on metacognitive judgments. For instance, if the confidence variable has access to the same information as the decision variable, then there is nothing more to learn from the identity of the action. This is the case in the postdecisional model shown in Figure 1B—the confidence variable is determined by the sum of pre- and postdecision evidence (equivalent to accumulating log-odds correct), and the action provides no further information beyond that provided by the predecision evidence (formally, *d* is conditionally independent of *a* given *X_conf_*). However, even in these cases of sequential evidence accumulation, effects of action may be obtained in practice. For instance, if the influence of predecision evidence decays over time, this would weaken the cross-talk between the decision and confidence variables, and actions would again carry weight when inferring the world state. In other words, if I make a perceptual decision based on some sensory evidence, but then go on to forget this evidence at a later point in time, I am left with only my decision when inferring what the world state might have been. Interestingly empirical data are potentially consistent with this prediction. Jazayeri and Movshon (2007) found that estimates of the direction of a random dot motion stimulus were biased in the direction of a previous binary choice. Such effects may be consistent with rational inference on possible world states in the face of imperfect integration or the inevitable decay of sensory evidence over time (Stocker & Simoncelli, 2008).

More broadly, the influence of one’s own actions on self-evaluation dovetails with the proposal that preferences and beliefs are constructed rather than revealed by judgments and decisions (Lichtenstein & Slovic, 2006). Postchoice preference change occurs when subjects increase their estimate of the value of an object after choosing it, while simultaneously decreasing the values of rejected items (Brehm, 1956; Sharot, De Martino, & Dolan, 2009). Although this phenomenon is famously theorized to result from subjects’ attempts to reduce cognitive dissonance, it can also be viewed in terms of rational inference in a model analogous to ours. Akin to perceptual categories, choice values are not perfectly known to the subject, but are probabilistic (De Martino et al., 2013; Lebreton et al., 2015; McFadden, 1980). To the extent that a subject’s reports reflect posterior beliefs about the value of the items, it becomes rational to incorporate one’s own actions if one has limited access to the decision variable underpinning choice, thereby leading to boosts in valuation after an object is chosen.

### Neural Implementation of Metacognition

The models considered here suggest an organizing framework for nascent findings on the neural basis of confidence and self-evaluation. In particular, correlates of confidence should be found across multiple putative internal states, including both those directly supporting actions and those supporting confidence ratings. Empirical studies in humans and nonhuman primates show that neural precursors of a decision are modulated by the eventual degree of confidence of the subject (Gherman & Philiastides, 2015; Kiani & Shadlen, 2009; Komura et al., 2013; Zizlsperger et al., 2014), and microstimulation of neurons encoding sensory evidence leads to biases in both choices and confidence ratings (Fetsch, Kiani, Newsome, & Shadlen, 2014). However, while confidence may covary with the activity of putative decision variables, the current framework predicts that metacognitive reports of confidence will critically depend on additional correlated states. Indeed, the mere fact that one brain area may “read-out” the decision variable from upstream neural populations may lead to a natural separation between decision and confidence variables. A study by Komura and colleagues is consistent with this proposal. In a motion discrimination task, the firing rate of pulvinar neurons correlated with the likelihood the monkey would choose an opt-out response. Inactivation of these neurons with muscimol led to an increase in opt-out responses without affecting first-order decision performance, as if the monkey lost confidence in its decision (Komura et al., 2013). This is potentially consistent with a confidence variable being encoded in cortico-thalamic loops (Kanai, Komura, Shipp, & Friston, 2015), and similar findings have been obtained through OFC inactivation in rodents (Lak et al., 2014).

A related line of work has identified a central role for the human prefrontal cortex (PFC) in metacognition (see Fleming & Dolan, 2012 for a review). Damage to the PFC leads to deficits in self-evaluation and impairments on a variety of tasks taxing metacognition (Pannu & Kaszniak, 2005; Schmitz & Johnson, 2007; Schnyer et al., 2004). Crucially these deficits may manifest in the absence of any changes in first-order performance: for instance, applying repetitive transcranial magnetic stimulation to the dorsolateral PFC in humans alters confidence but not performance in a visual discrimination task (Rounis et al., 2010), and patients with lesions to anterior sectors of the PFC show a reduced correspondence between confidence and accuracy (reduced Type II ROC area) on a perceptual task despite performance remaining unaffected (Fleming et al., 2014). In addition, studies using functional imaging in humans and single-unit recording in nonhuman primates and rodents have identified correlates of confidence in prefrontal cortex and interconnected subcortical regions (De Martino et al., 2013; Fleming et al., 2012; Hebart, Schriever, Donner, & Haynes, 2016; Hilgenstock, Weiss, & Witte, 2014; Kepecs et al., 2008; Lak et al., 2014; Middlebrooks & Sommer, 2012). In relation to the current framework, these findings may be consistent with prefrontal involvement in representing a confidence variable and/or hyperparameters about sources of decision uncertainty (Lau, 2008), and/or in representing the output of a confidence computation for subsequent report (Fleming & Dolan, 2012).

Second-order computation requires integration of state information (e.g., *X_conf_*) with knowledge about the selected action. Importantly this convergence should be flexible and domain-general.^4^ Consider a task where auditory stimuli are arbitrarily mapped to eye movements, and visual stimuli to hand movements. To compute confidence in the model in Figure 1C one would need to combine information about each sensory modality with corollary discharge (or proprioceptive feedback) from the relevant motor system. One solution to this problem would be to maintain global representations of sensory evidence in a response-independent frame of reference (Heekeren, Marrett, Ruff, Bandettini, & Ungerleider, 2006; Ho, Brown, & Serences, 2009; O’Connell, Dockree, & Kelly, 2012; Tosoni, Galati, Romani, & Corbetta, 2008). The frontopolar cortex (FPC; Brodmann area 10) in primates is one potential convergence zone for integrating state and action information in the service of second-order computation. The FPC receives multimodal inputs from higher-order sensory and motor regions in the parietal, frontal, and temporal lobes (Burman, Reser, Yu, & Rosa, 2011; Neubert, Mars, Thomas, Sallet, & Rushworth, 2014; Ramnani & Owen, 2004), and convergent evidence supports its role in human metacognition (Baird, Smallwood, Gorgolewski, & Margulies, 2013; De Martino et al., 2013; Del Cul et al., 2009; Fleming et al., 2010, 2012; 2014; Hilgenstock et al., 2014; McCurdy et al., 2013; Miele, Wager, Mitchell, & Metcalfe, 2011; Yokoyama et al., 2010). One study in monkeys shows that FPC neurons code the chosen response at the time of feedback in a decision task, but do so differentially depending on whether the response was correct or erroneous. Critically these signatures emerge before external feedback is given, potentially consistent with an evaluation of whether the action taken was appropriate (Tsujimoto, Genovesio, & Wise, 2010, 2011). Another candidate neural nexus for state-action integration is the dorsomedial prefrontal cortex (dmPFC; encompassing the paracingulate cortex and pre-supplementary motor area). Studies of error detection observe increased activity in dmPFC when errors are made on simple choice reaction time (RT) tasks in the absence of external feedback (Carter et al., 1998; Dehaene et al., 1994; Gehring et al., 1993), and the dmPFC is in turn interconnected with insula and FPC, suggesting a possible circuit for metacognitive evaluation (Baird et al., 2013; Hilgenstock et al., 2014).

Finally, the model of metacognition we outline here has much in common with schemes for recursive inference in social cognition (Goodman & Baker, 2009; Shafto, Goodman, & Frank, 2012). Confidence is formed through second-order evaluation of a coupled but distinct decision system, computationally equivalent to inferring the performance of another actor. While here we have focused on the implications of this framework for self-directed metacognition, to the extent that self- and other-evaluation rely on common mechanisms, brain networks previously linked to theory of mind (ToM) may also play a role in metacognition (Carruthers, 2009). Previous studies have identified similarities in neural activity for self- and other-judgments (Decety & Sommerville, 2003; C. D. Frith & Frith, 1999; Jenkins et al., 2008; Mitchell, Banaji, & Macrae, 2006) albeit with a focus on personal-level judgments about beliefs, attitudes or personality characteristics. It will be of interest to determine whether these ToM networks are additionally recruited when inferring subpersonal states such as one’s confidence in percepts or memories.

### Relationship Between Metacognitive Monitoring and Control

Computing confidence in a decision is a type of metacognitive monitoring, and may be distinct from processes supporting metacognitive control (Nelson & Narens, 1990). However, accurately inferring one’s confidence in a task is important for the future control of behavior. For instance, a child studying for an exam will perform better if they have an accurate impression of how much there is still to learn (Veenman et al., 2004). In the absence of external feedback, such estimates may be furnished by second-order computation, which outputs a subjective probability of success. This probability provides a useful indicator of whether a previous decision should be corrected (Resulaj et al., 2009), whether a subsequent step in a chain of decisions should be initiated (Dehaene & Sigman, 2012), whether to make the task easier by offloading intentions into the environment (Gilbert, 2015), or more generally when it is advantageous to deliberate (Keramati et al., 2011) or engage cognitive control (Boureau et al., 2015; Shenhav, Botvinick, & Cohen, 2013). Here we focus on the generation of confidence in a single task, but one could envisage replicating this architecture to maintain internal estimates of long-run confidence over a number of tasks (Donoso, Collins, & Koechlin, 2014). We would therefore predict a close relationship between metacognitive estimates of confidence and the strategic control of decision-making.

### Metacognition and Clinical Insight

A common factor in a range of neurological and psychiatric disorders is a loss of insight (David et al., 2012)—the ability to recognize and describe one’s own behavior, cognition, and mental states. For instance, a patient with addiction may not recognize a need for treatment due to impaired insight into his or her addictive behaviors (Goldstein et al., 2009), consistent with impairments of metacognitive sensitivity in this population (Moeller et al., 2016). Deficits in metacognitive sensitivity have also been documented in pathological gambling (Brevers et al., 2014) and brain injury (Fleming et al., 2014; Ham et al., 2014; Pannu & Kaszniak, 2005), and have been suggested to underpin a variety of impairments in schizophrenia, ADHD and anosagnosia (Klein et al., 2013). Second-order computation provides a possible framework within which to understand such deficits. For instance, loss of insight may correspond to a pathologically increased coupling between internal states, reducing the ability for error detection (Figure 4D), a reduction in the precision of the confidence variable (Figure 8A), aberrant beliefs (hyperparameters) about different sources of uncertainty (see Figure 9), or any combination of these factors. Actions would occur but the subject would have little knowledge of *why* they occurred, or whether they were appropriate for the current situation. Restoring insight in such cases may therefore be aided by a better understanding of the computational and neural basis of metacognition.

### Limitations and Future Directions

We have focused on modeling a two-choice perceptual discrimination for computational simplicity. However, the key feature of the model is qualitative—second-order states are harnessed to infer confidence in first-order decisions. This holds promise for generalizing the framework to other domains, such as memory- or value-based choices. In addition, we have not considered the role of learning or prior beliefs about the task structure in constructing self-evaluations. For instance, expectations about possible world states **(***P*(*d*)) should influence the computation of confidence (Sherman, Seth, Barrett, & Kanai, 2015). We have also not touched upon how subjects learn the model of the task in the first place (corresponding to reduction in uncertainty at the rule or strategy level, Bach & Dolan, 2012; Donoso et al., 2014) or learn beliefs (hyperparameters) about self-ability, but these are likely to be important for understanding the dynamics of self-evaluation over longer timescales. Moreover such learning is likely to be influenced by our interactions with other individuals, allowing coordination of confidence at the group level (Bahrami et al., 2012; Shea et al., 2014).

In many laboratory decision-making tasks (and in the simulations carried out here), actions are binary, such as a button press or eye movement. In practice, however, even simple actions are constructed by specifying the kinematics and forces needed to produce a particular motor output. Indeed, individuals have been shown to take action kinematics into consideration when judging the confidence of another individual (Patel et al., 2012), and the specifics of action planning impacts upon error-related brain activity (Bernstein, Scheffers, & Coles, 1995; Torrecillos, Albouy, Brochier, & Malfait, 2014). An interesting avenue for future investigation is the extent to which this richness of action specification is incorporated into decision confidence, and how this information is routed to metacognitive computations.

Finally, as touched upon above, our model is situated at the computational level (Marr, 1982) and remains agnostic about algorithmic or mechanistic implementation. Future efforts could harness our framework to guide construction of finer-grained Bayesian models incorporating temporal dynamics or candidate neural network implementations (Beck et al., 2008; Fiser, Berkes, Orbán, & Lengyel, 2010; Insabato et al., 2010; Ma & Jazayeri, 2014; Pasquali et al., 2010; Rao, 2004).

### Conclusions

The model outlined in this paper casts self-evaluation as a second-order inference on the efficacy of one’s own behavior. Such a model has the potential to provide common ground for comparing data from different paradigms such as confidence and error detection, and provides a normative framework for understanding a range of dissociations between metacognition and performance. In addition, it predicts a novel role for actions in contributing to estimates of decision confidence. We have outlined the implications of second-order computation for behavioral control and for candidate neurobiological implementations of metacognition. We hope this will provide a conceptual and theoretical framework for studies of metacognitive computation, and motivate a number of empirical hypotheses to be tested in future research.

## Figures and Tables

**Table 1 tbl1:** Summary of Model Variants and Their Ability to Accommodate Qualitative Features of Empirical Data

Variant	First-order	Post-decisional	Second-order
X-pattern in confidence	Yes	Yes	Yes
Error detection	No	Yes	Yes
Effects of choice on confidence	No	No	Yes

## Footnotes

1 We do not explicitly consider the within-trial dynamics of the decision variable here though we appreciate their importance for a complete account of confidence (Fetsch et al., 2015). Just as sequential sampling models represent dynamic extensions of signal detection theory (Gold & Shadlen, 2002; Link & Heath, 1975; Pleskac & Busemeyer, 2010; Ratcliff, 1978), the framework we consider here may be naturally extended to incorporate sequential samples of evidence. Because the primary aim of this paper is to contrast first- and second-order computation, we restrict ourselves to the simpler, static cases, returning in the Discussion to consider the issue of dynamics in greater detail.

2 Kepecs and colleagues have shown that spike rates of single neurons in orbitofrontal cortex, a putative neural correlate of confidence, show an X-pattern as a function of external stimulus strength (Kepecs et al., 2008). Given that the internal state of a first-order model does not show this pattern, it is tempting to instead conclude that a more complex model is needed to account for these findings. However, this conclusion does not necessarily follow: again, the experimenter has access only to stimulus strength rather than the animal’s decision variable, and similar considerations apply as when interpreting behavioural data.

3 The related terms resolution and calibration are often employed in studies of probability judgments.

4 Similarly Timmermans et al. (2012) point out that metacognition “requires that the first-order representations that are responsible for performance be accessed in a manner that is independent from their expression in behaviour” (p. 1416).

## A Derivation of Second-Order Confidence

The second-order model posits that the decision and confidence variables are draws from a multivariate Gaussian with covariance matrix ∑:

$$\left[\begin{array}{c} X_{act}\\ X_{conf} \end{array}\right]\;∼\;N(d,\sum )$$

$$\sum =\left[\begin{array}{cc} \sigma_{act}^{2} & \rho \sigma_{act}\sigma_{conf}\\ \rho \sigma_{act}\sigma_{conf} & \sigma_{conf}^{2} \end{array}\right]$$

To compute confidence, *z*, the observer infers (for the purpose of marginalizing) the state of the decision variable driving choice (*X_act_*) from the confidence variable (*X_conf_*).

$$z=P\left(a=d|X_{conf},a,\sum \right)=\{\begin{array}{c} P\left(d=1|X_{conf},\;a,\sum \right)\;if\;a=1\\ 1-P\left(d=1|X_{conf},\;a,\sum \right)\;if\;a=-1\; \end{array}$$

In the following we unpack computation of *P*(*d*|*X_conf_*, *a*, ∑), suppressing covariance parameters ∑ for clarity. As in the first-order model, confidence depends on the posterior over *d* computed using Bayes rule:

$$P\left(d|X_{conf},a\right)=\frac{P(d|X_{conf})P\left(a|X_{conf},d\right)}{\sum_{d}P\left(d|X_{conf}\right)P\left(a|X_{conf},d\right)}$$

Starting with the second term, *P*(*a*|*X_conf_*, *d*):

$$P\left(a|X_{conf},d\right)=\int P\left(a|X_{act}\right)P(X_{act}|X_{conf},d)\;dX_{act}$$

$$=\overset{\infty }{\underset{0}{\int }}P\left(X_{act}|X_{conf},d\right)\;dX_{act}\;\text{if}\;a=1$$

$$\;=\overset{0}{\underset{-\infty }{\int }}P\left(X_{act}|X_{conf},d\right)\;dX_{act}\;\text{if}\;a=-1$$

where the latter two expressions are due to the threshold response rule, *a* = 1 whenever *X_act_* > 0. This expression is a cumulative density function of the conditional density of a multivariate Gaussian, which itself is a univariate Gaussian with the following mean and standard deviation:

$$P\left(X_{act}|X_{conf},d\right)\;∼\;N(\mu_{X_{act}|X_{conf}},\sigma_{X_{act}|X_{conf}})$$

where $\mu_{X_{act}|X_{conf}}=d+\frac{\sigma_{act}}{\sigma_{conf}}\rho \left(X_{conf}-d\right)$

$$\sigma_{X_{act}|X_{conf}}=\sqrt{\left(1-\rho^{2}\right)\sigma_{act}^{2}}$$

The first term is the normalized likelihood of *X_conf_* given *d*:

$$P\left(d|X_{conf}\right)=\frac{P\left(X_{conf}|d\right)}{\sum_{d}P\left(X_{conf}|d\right)}$$

i.e., Bayes’ rule with the uniform prior *P*(*d*) canceled, where *P*(*X_conf_*|*d*) = ϕ(*X_conf_*, *d*, σ_*conf*_) and ϕ () is the standard Gaussian density function:

$$\phi \left(x,\mu ,\;\sigma \right)=\frac{1}{\sigma \sqrt{2\pi }}e^{\frac{-{\left(x-\mu \right)}^{2}}{2\sigma^{2}}}$$

## B Simulation Details

### Internal Representations Supporting Decision Confidence

To produce the plots in Figure 3 we simulated 10,000 trials at each of 7 levels of stimulus strength θ = [0 0.032 0.064 0.128 0.256 0.512 1.0]. For the first-order and postdecisional models σ = 1. For the second-order model, parameter settings were σ_*act*_ = 1, σ_*conf*_ = 0.5 and ρ = 0.4. Confidence was sorted according to whether the model’s response was correct or incorrect. For all models we also binned confidence into tertiles of the unsigned confidence variable |*X_conf_*|.

### Internal Representations Supporting Error Monitoring

For each cell of the second-order model parameter grid in Figure 4D we simulated 10,000 trials and recorded the proportion of errors that were detected (errors with confidence levels of less than 0.5). σ_*act*_ (and therefore the objective error rate) was kept constant.

### Influence of Actions on Confidence

To examine the effects of actions on subsequent ratings we simulated two conditions, “rate-choose” and “choose-rate” for both the first- and second-order models. Confidence in the rate-choose condition was defined as the posterior probability of a future decision being correct (the max over possible actions; Kvam et al., 2015):

$$\text{confidence}=\text{max}[P\left(d=1|X_{conf}\right)\;P\left(d=-1|X_{conf}\right)]$$

To create Figure 6B and 6C we simulated 10,000 trials at each of 7 levels of stimulus strength θ = [0 0.032 0.064 0.128 0.256 0.512 1.0] with σ_*act*_ = 1, σ_*conf*_ = 1 and ρ = 0.6. To determine the choice-dependence of bias and metacognitive sensitivity on second-order model parameters we simulated 10,000 trials at a single level of stimulus strength θ = 1 while varying ρ and σ_*conf*_. σ_*act*_ was fixed at 1, ensuring constant performance. ρ varied across 10 levels equally spaced between 0.1 and 0.9 while keeping σ_*conf*_ fixed at 1; σ_*conf*_ varied across 10 levels equally spaced between 0.5 and 1.5 while keeping ρ fixed at 0.6. Bias was calculated as the mean confidence level collapsing across correct and error trials; metacognitive sensitivity was calculated as the difference between mean confidence on correct and incorrect trials.

### Modeling Dissociations Between Performance and Confidence

Type II ROCs were plotted by sweeping confidence criteria across 20 evenly spaced steps from 0 to 1 and calculating Type II hit rates (the proportion of high confidence trials when the model is correct) and false alarm rates (the proportion of high confidence trials when the model is incorrect; see Fleming & Lau, 2014; Galvin et al., 2003 for further details); 10,000 trials were simulated at each parameter setting.

To construct Figure 8C and 8D, 100 data sets were simulated each containing 1000 trials. σ_*conf*_ and σ_*act*_ were each generated from independent uniform random draws on the interval [1.5 2.5]. For both simulated and empirical data sets, meta-*d*′ was fit using maximum likelihood methods instantiated in the code provided by Maniscalco & Lau (www.columbia.edu/~bsm2105/Type2sdt/).

The data sets contributing to Figure 8E have been published in full elsewhere (Fleming et al., 2010, 2012; E. C. Palmer et al., 2014; L. G. Weil et al., 2013). Briefly, each study administered a perceptual decision task with trial-by-trial confidence ratings elicited postdecision on an arbitrary numerical scale ranging from 1 to 6. The number of trials available for analysis ranged from 250 to 500 per subject. In all studies, task difficulty was controlled by a one-up two-down staircase that targeted a performance level of approximately 71% correct. Three of the four studies employed a 2-interval forced choice detection task in which subjects were asked to report which interval contained a pop-out Gabor patch (Fleming et al., 2010; E. C. Palmer et al., 2014; L. G. Weil et al., 2013); one study employed a face/house discrimination task (Fleming et al., 2012).
